# Synthesis and redox behavior of new ferrocene-π-extended-dithiafulvalenes: An approach for anticipated organic conductors

**DOI:** 10.3762/bjoc.5.6

**Published:** 2009-02-19

**Authors:** Abdelwareth A O Sarhan, Omar F Mohammed, Taeko Izumi

**Affiliations:** 1 Chemistry Department, Faculty of Science, Assiut University, Assiut 71516, Egypt; 2 Department of Chemistry and Chemical Engineering, Faculty of Engineering, Yamagata University, 3-16 Jonan 4-Chome, Yonezawa 992-8510, Japan

**Keywords:** cyclic voltammetry, charge-transfer (CT) complexes, diacylferrocenes, electrochemical properties, ferrocene-π-extended-dithiafulvalenes [bis(1,3-DTF)Fc’s], organic conductors

## Abstract

A number of new ferrocene-π-extended-dithiafulvalenes were successfully synthesized as new electron donor compounds. The chemical structures and electrochemical behaviors of these compounds were investigated using several spectroscopic methods. The synthesis of these compounds was achieved using the modified Wittig–Horner cross-coupling reaction using *n*-BuLi/THF at temperature varies from −78 °C to 0 °C. These new classes of bis(1,3-dithiafulvalene)ferrocenes have the 1,3-dithiole ring system separated by ferrocene as conjugated spacer. The ferrocene-dithiafulvalenes derivatives **9** and **12** were prepared as side products during the synthesis of the targeted compounds as bis(1,3-dithiafulvalene)ferrocenes **8**, **10** and **11** in variable yields. The redox properties of the compounds have been investigated by cyclic voltammetry at ambient temperature using tetra-*n*-butylammonium perchlorate (TBAP) as the supporting electrolyte compared to ferrocene and the derivative **9**. In CH_2_Cl_2_ on a Pt working electrode and at ambient temperature, two oxidation waves associated with two reduction waves at scan rates 100 mV s^−1^ were observed for **9** and **12**. In contrast the anodic peak potential of bis(1,3-dithiafulvalene)ferrocenes **8**, **10** and **11** exhibited two and three oxidation waves associated with two reduction waves.

## Introduction

Electron-transfer reactions have found highly useful applications for organic synthesis, and therefore have encouraged scientists to synthesize a wide variety of molecules to develop new conducting charge transfer complexes [1–3]. As a result, we have turned our attention to the synthesis of a new series of tetrathiafulvalene derivatives. These are particularly intriguing molecules for several reasons: (i) they are chemically and photochemically stable in solution; (ii) they exhibit an extended conjugated structure which provides strong absorption in the UV/vis regions; (iii) they are considered as excellent building blocks for intramolecular charge and photoinduced electron-transfer processes [4]; (iv) they can be oxidized successively and reversibly (multistage redox states) to the cation radical and dication species. Related to this issue, Hudhomme and co-workers have synthesized and characterized a donor-acceptor dyad system involving tetrathiafulvalene (TTF) as electron donor attached by a flexible spacer to perylene derivatives as electron acceptor [5]. They have shown that the fluorescence of the tetrathiafulvalene–perylene derived dyad can be reversibly modulated by the transformation of the TTF unit into its radical cation and dication.

After organic chemists had discovered organic metal tetrathiafulvalene-tetracyanoquinodimethane (TTF-TCNQ) charge transfer complexes, various synthetic approaches to tetrathiafulvalenes gained wide attention, and charge-transfer from TTF to TCNQ was extensively recognized [6–7]. For instance, π-extended-dithiafulvalenes have been successfully used as multi-electron donor moieties with high electrical conductivities in the preparation of new charge transfer (CT) complexes [8–9]. However, a number of modifications of the TTF framework have been added to improve their electrical conductance properties. For instance, TTFs containing two or more fused or covalently attached TTF units have been used for preparing superconducting salts [10–11]. Recently, a number of modifications have been performed even on the tetrathiafulvalene (TTF) skeleton **1** in the search of new molecular-based organic metals [12–16]. Furthermore, the extension of conjugation between the two 1,3-dithiole units of TTF and their conducting salts has been prepared studied [17–23].

**Figure 1:**
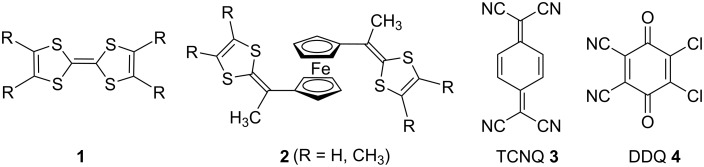
Donors and acceptor compounds.

The first compound belonging to the class of 1,1′-bis[(1,3-dithiol-2-ylidene)alkyl]ferrocenes (**2**) was shown to form 1:1 CT complexes with tetracyano-*p*-quinodimethane (TCNQ, **3**) and dichlorodicyanoquinone (DDQ) **4**, see Figure 1 [24–26]. Subsequently, a number of reports have appeared dealing with the electrochemical properties of these CT complexes of tetrathiafulvalenes having a ferrocene moiety [10–11].

These findings prompted us to synthesize and investigate the electrochemical properties of symmetrical 1,1′-bis[(1,3-dithiol-2-ylidene)heteroaryl/aryl]ferrocenes substituted with heterocyclic and aromatic ring moieties separated by ferrocene moiety as the conjugated spacer. Femtosecond time-resolved absorption and Raman studies are in progress to aid in the understanding of the photophysical properties of the bis(1,3-DTF)Fc-TCNQ charge transfer complexes, trying to pick up the transient of the mono- and dication and anion radical of these systems with TCNQ, respectively. From this study we can estimate the timescales of the charge separation and recombination of the produced radical ion pairs. Such laser spectroscopic studies will have a big impact in future, leading not only new synthetic applications, but also to the discovery of new photophysical properties of these systems [27].

## Results and Discussion

In this work we synthesized a series of novel 1,1′-bis[(1,3-dithiol-2-ylidene)heteroaryl/aryl]ferrocene [1,1′-bis(1,3-DTF)Fc] derivatives as new electron donor compounds using the direct Wittig–Horner cross coupling reaction. The starting 1,1′-diacylferrocene derivatives **5a** and **5b** were synthesized cleanly and in high yields according to previously reported methods [12–13,15]. The 1,3-benzodithiole-2-phosphonate **6** was obtained in relatively high yield as described previously in literature [14,28–29].

**Scheme 1:**
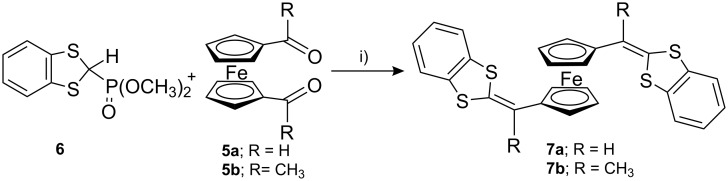
Synthesis of π-extended dithiafulvalenes **7a** and **7b**. i) *n*-BuLi/THF, −78 °C, 15 min, then rt, overnight.

Reaction of ferrocene-1,1′-dicarboxaldehyde (**5a**) or 1,1′-diacetylferrocene (**5b**) with 1,3-benzodithiole-2-phosphonate (**6**) in dry THF in the presence of *n*-BuLi at −78 °C following the Wittig–Horner reaction method afforded the corresponding 1,1′-bis[(1,3-dithiol-2-ylidene)methyl]ferrocenes **7a** and **7b** in good yields (Scheme 1). The 1,1′-bis(1,3-dithiafulvalene)ferrocene **7a** was prepared following the procedures reported by A. Togni et al. [30] as dark red crystals in good yield, while **7b** was obtained according to procedures similar to those of Sarhan et al. [31].

**Scheme 2:**
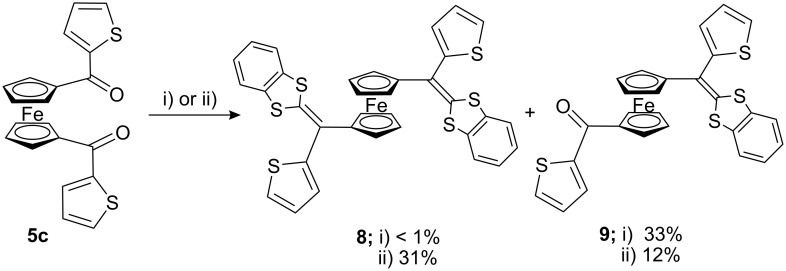
Synthesis of π-extended dithiafulvalenes **8** and **9**. i) **6**, *n*-BuLi, THF, −78 °C, 15 min; then, **5c**, −78 to 20 °C, overnight. ii) **6**, *n*-BuLi, THF, −78 °C, 15 min; then **5c**, −20 to 0 °C, 15 min, then rt overnight.

On application of the Wittig–Horner reaction (*n*-BuLi, −78 °C, THF) to 1,1′-bis(2-thienoyl)ferrocene (**5c**), with 1,3-benzodithiole **6** the unexpected ferrocene-dithiafulvalene (Fc-DTF) **9** was obtained as an orange-red oil in 33% yield. The targeted 1,1′-bis(1,3-DTF)Fc **8** was obtained in very low yield (<1%), could not be isolated in pure form and could only be detected by FAB mass spectra (M^+^ 678) (Scheme 2) [13]. However, upon reaction of the diacylferrocene **5c** with the 1,3-benzodithiole-2-phosphonate **6** using a slight modification of the Wittig–Horner procedures, by carrying out the reaction at −20 to 0 °C and subsequently purifying by column chromatography using chloroform/hexane mixture, the 1,1′-bis(1,3-DTF)Fc **8** was obtained as dark red crystals in 31% yield followed by **9** in 12% yield in the second fraction.

**Scheme 3:**
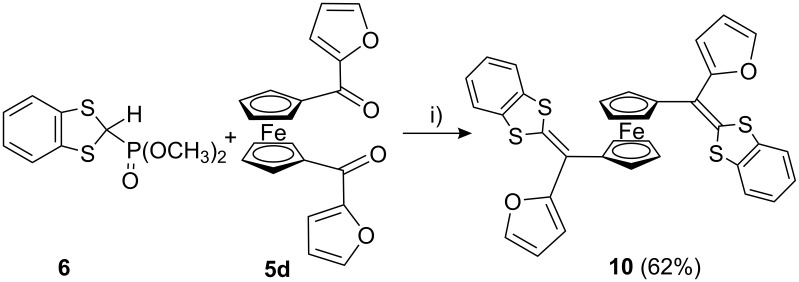
Synthesis of π-extended dithiafulvalenes **10**. i) **6**, *n*-BuLi, THF, −78 °C, 15 min; then **5d**, −20 to 0 °C, 15 min, then rt overnight.

Similarly, when 1,1′-bis(2-furoyl)ferrocene (**5d**) was subjected to reaction with **6** under the modified Wittig–Horner reaction at −20 to 0 °C in the presence of *n*-BuLi/THF followed by stirring the reaction mixture under nitrogen atmosphere overnight, the 1,1′-bis(1,3-DTF)Fc **10** was obtained as dark red crystals in 62% yield (Scheme 3).

**Scheme 4:**
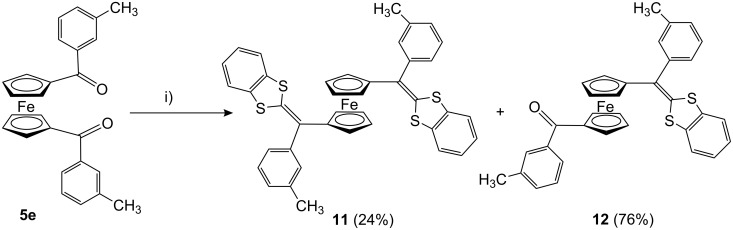
Synthesis of π-extended dithiafulvalenes **11** and dithiafulvalene **12**. i) **6**, *n*-BuLi, THF, −78 °C, 15 min; then **5e**, −20 to 0 °C, 15 min, then rt overnight.

Moreover, when this reaction was carried out allowing 1,1′-bis(*m*-tolylcarbonyl)ferrocene (**5e**) to react with **6** following the same modified Wittig–Horner procedure, the product was identified as 1,1′-bis(1,3-DTF)Fc **11** in 24% yield in addition to the (1,3-DTF)Fc **12** as a major product (76% isolated yield) (Scheme 4).

### Electrochemistry

The electrochemical redox properties of the newly synthesized Fc-DTFs **9** and **12**, the 1,1′-bis(1,3-DTF)Fc’s **8**, **10**, **11**, and the acylferrocenes **5c**–**e** were studied by cyclic voltammetry at room temperature in dry CH_2_Cl_2_ solutions, using Pt working electrode, Pt gauze as a counter electrode and Ag/AgCl as a reference electrode and tetra-*n*-butylammonium perchlorate (TBAP) as the supporting electrolyte. The electrochemical data for the investigated compounds were compared to those of ferrocene and summarized in Table 1, Table 2 and Table 3. The data show that compounds **5a**–**5e** have more positive redox values than ferrocene.

**Table 1:** Cyclic voltammetric parameters of the compounds **5a**–**e** (5 × 10^−4^ mol/L) and ferrocene Fc on a Pt working electrode, Pt gauze counter electrode and Ag/AgCl reference electrode in dry CH_2_Cl_2_ at ambient temperature using TBAP 0.1 mol L^−1^ concentration as the supporting electrolyte, scan rate 100 mV s^−1^

Compound No.	*E*_pc_/mV	*E*_pa_/mV	*E*^0′^/mV	Δ*E*_p_/mV

**Fc**	481	554	518	73
**5a**	1047	1133	1090	86
**5b**	985	1075	1030	90
**5c**	960	1094	1027	134
**5d**	901	1015	958	114
**5e**	913	1028	972	115

**Table 2:** Cyclic voltammetric parameters of the compounds Fc-DTF **9** and **12** (5 × 10^−4^ mol/L) on a Pt working electrode, Pt gauze counter electrode and Ag/AgCl reference electrode in dry CH_2_Cl_2_ at ambient temperature using TBAP 0.1 mol L^−1^ concentration as the supporting electrolyte, scan rate 100 mV s^−1^

Compound No.	*E*_pc_/mV		*E*_pa_/mV		*E*^0′^/mV		Δ*E*_p_/mV	
	P_1_	P_2_	P_1_	P_2_	P_1_	P_2_	P_1_	P_2_

Fc-DTF **9**^a^	653	982	725	1119	689	1051	72	137
Fc-DTF **12**	600	977	698	1158	649	1068	98	181

^a^Fc-DTF **9** was measured at scan rate 20 mV s^−1^.

**Table 3:** Cyclic voltammetric parameters of the compounds **8**, **10**, **11** (5 × 10^−4^ mol/L) on a Pt working electrode, Pt gauze counter electrode and Ag/AgCl reference electrode in dry CH_2_Cl_2_ at ambient temperature using TBAP 0.1 mol L^−1^ concentration as the supporting electrolyte, scan rate 100 mV s^−1^

Compound No.	*E*_pc_/mV			*E*_pa_/mV			*E*^0′^/mV			Δ*E*_p_/mV		
	P_1_	P_2_	P_3_	P_1_	P_2_	P_3_	P_1_	P_2_	P_3_	P_1_	P_2_	P_3_

**8** (SR = 20 mV)	414	925		501	1038		458	982		87	113	
**8** (SR = 100 mV)	394	832		570	905	1043	482	869	938	176	73	211
**10**	449	721	992	519	889	1067	484	805	1030	70	168	75
**11**	258	859		343	1022		258	941		85	163	

These results indicate that the separation of the anodic and the cathodic peak potentials, Δ*E*_p_, are almost the same for compounds **5d** and **5e**, while a small positive shift in the formal potential, *E*^0′^, (60 mV) for compound **5a** was observed compared to compound **5b**. This shift can be attributed to the replacement of hydrogen atom in **5a** by the electron donating methyl group in **5b**, which facilitates the redox process of compound **5b** [32]. On the other hand, on replacement of the hydrogen atom in **5a** with conjugated ring system, e.g. thiophene, furyl or *m*-tolyl, a decrease in the formal potential, *E*^0′^, at 63, 118 and 133 mV is observed for compounds **5c**, **5e** and **5d**, respectively. This indicates that the substitution of the hydrogen atom with a conjugated ring system leads to a decrease in the formal potential, *E*^0′^. The Δ*E*_p_ values are 86, **5a**; 90, **5b**; 134, **5c**; 114, **5d**; 115, **5e**; mV compared to ferrocene 73 mV. The cyclic voltammetric behavior of compounds **5d** and **5e** is almost the same, in which Δ*E*_p_ values are 114 mV and 115 mV for **5d** and **5e** respectively, whereas a positive shift of 14 mV in the *E*^0′^ was observed for compounds **5e** compared to compound **5d**. This confirms that the substituted tolyl ring in compound **5e** enhances the electron transfer process and consequently its redox behavior. Compared to the parent ferrocene a large positive shift in the formal potential, *E*^0′^, (572, 512, 509, 440, 454 mV) was observed compared to compounds **5a**–**e** respectively.

The electrochemical behaviors of diacylferrocene derivatives **5a**–**e** are markedly affected by the scan rate, in which at higher scan rate (ν ≥ 600 mV s^−1^), broadening of Δ*E*_p_ was observed (Δ*E*_p_ > 230 mV), indicating that the irreversibility of the electron-transfer process was maintained under these conditions, possibly due to the onset of kinetic complications. The electrochemical redox properties of compounds **5a**–**e** were studied by cyclic voltammetry and the data are listed in Table 1. One oxidation wave potential associated with one reduction wave for these ferrocenyl diketones was observed in the potential range ca 1015–1100 mV at lower scan rate (10–600 mV s^−1^) (Table 1).

Compounds **9** and **12** also showed some common features depending the solvent and scan rate effects. In CH_2_Cl_2_ on a Pt electrode and at ambient temperature, compounds **9** and **12** showed two oxidation waves with peak potentials of 725, 1119 mV for **9** and 698, 1158 mV for **12** at scan rates 100 mV s^−1^. For **9** and **12**, such a process is electrochemically reversible or quasi-reversible (Δ*E*p^1^ 72 mV and Δ*E*p^2^ 137 mV) for **9** and (Δ*E*p^1^ 98 mV and Δ*E*p^2^ 181 mV) for **12**. The Δ*E*p^2^ was not observed with an increase of scan rate and the second wave was distorted when increasing the scan rate more than 100–400 mV s^−1^ (Table 2).

The 1,1′-bis(1,3-DTF)Fc’s **8**, **10** and **11** showed three couples of redox waves observed clearly in the cyclic voltammograms at the potential range, *E*_pa_ ca 340–1070 mV at lower scan rate (10–100 mV s^−1^). The first couple of redox waves in the potential range ca 340–725 mV is due to the redox process of the DTF/DTF^+^ system, whereas the second couple of redox waves in the potential range ca. 900–1022 mV is attributed to the Fc/Fc^+^ redox process. The third couple of redox waves which attributed to the DTF^+^/DTF^++^ appeared at 1040–1070 mV, Table 3.

**Figure 2:**
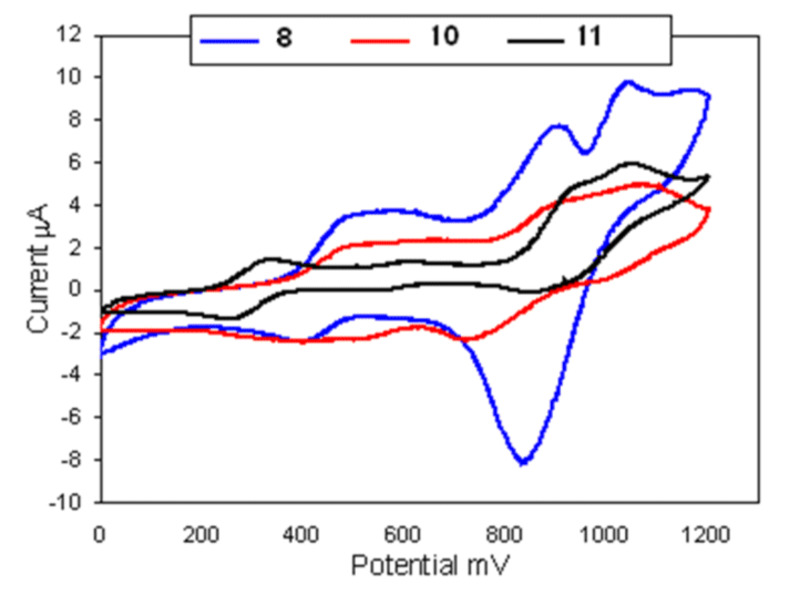
Cyclic voltammetry (CV) of compounds **8**, **10** and **11** in CH_2_Cl_2_ at scan rate 100 mV.

The redox behavior of compound **8** was studied using different scan rates (10, 20, 50, 200 and 300 mV s^−1^) at ambient temperature, and showed only the appearance of two quasi-reversible oxidation processes associated with two reduction waves (SR = 20 mV; *E*p^1^ = 414–501 mV, *E*p^2^ = 925–1035 mV) depending on the scan rates, Table 3. At scan rate SR = 100 mV (*E*p^1^ = 390–570 mV, *E*p^2^ = 832–905 mV and *E*_p_^3^ = 1043 mV) three oxidation processes with two associated reduction peaks could be observed. Controlled potential coulometry in correspondence to the first anodic step (*E*^Ox1^ = +0.57 V) consumes one electron/molecule typical of ferrocenium species, showing a cyclic voltammetric response quite complementary to the first step illustrated in Figure 2. The second oxidation step, although displaying a good extent of chemical reversibility in cyclic voltammetry and consuming one electron/molecule (*E*^Ox2^ = +0.905 V), affords a dirty green solution which no longer exhibits a cyclic voltammetric profile attributable to the generation of redox congeners of **8**. This proves that the primarily electro-generated dication [**8**]^+2^ is a transient species. This third anodic step is an irreversible oxidation at high-potential values (*E*_p_ = +0.938 V). These voltammetric data are in agreement with those previously reported [27,30] for the redox behavior of the compound **8** in which the third oxidation peak was observed as irreversible at a more positive potential value (*E*_p_^3^ = 1043 mV), Table 3 and Figure 2.

The aforementioned electrochemical results of compounds **7a**, **7b**, **8**, **10** and **11**, where ferrocene is a spacer between the two 1,3-dithiole units, indicate that the interaction of ferrocene directly with the DTF rings causes significant changes in electron donating properties and consequently the electrochemical behavior of these compounds. Comparing the data of compounds **8**, **10** and **11** with those previously prepared by Togni et al. [30] and Sarhan et al. [31] we found that the CV data obtained here was in agreement with that reported for **7a** and **7b**, while for compounds **8**, **10** and **11** three reversible oxidation processes associated with three reduction waves were clearly resolved. Controlled potential coulometry corresponding to the first anodic step for **8** (*E* = 570 mV) consumed one electron/molecule. We also found that the third anodic process is irreversible in character. Analysis of the cyclic voltammograms relevant to the first oxidation process with scan rates varying from 20 mV s^−1^ to 400 mV s^−1^ shows that the peak-to-peak separation progressively increases and the 100 mV scan rate is ideal in most cases. Introducing the conjugated ring system such as 2-thienyl, 2-furyl and C_6_H_4_CH_3_-*m* leads to the formation of good donor ability highly expected to form stable conducting materials, opening a very interesting research area in the ET reaction and intermolecular CT complexes.

## Conclusion

In conclusion, a number of 1,1′-substituted diacylferrocenes **5a**–**e** were synthesized by Friedel–Crafts reaction. Their structures were confirmed by spectral analyses and were in satisfactory agreements with those reported in literature. Some new 1,1′-bis(1,3-DTF)Fc’s and Fc-DTFs were made as multicomponent electron donor systems by the Wittig–Horner reaction of the respective phosphonate ester **6** with different ferreocenylketones using the modifications introduced onto the reaction. The redox chemistry of the ferreocenylketones and these new π-conjugated hybrids **8**, **10** and **11** has been studied using cyclic voltammetry at ambient temperature on a Pt working electrode, using TBAP as the supporting electrolyte. The CV exhibited good donor properties, showing a one-electron quasireversible oxidation potential. The oxidation potential values, and to a larger extent the reduction potential values, are strongly influenced by the scan rate. Increasing the scan rate from 20 to 600 mV s^−1^ leads to an increase of the oxidation potential values. The Fc-DTF derivatives **9** and **12** were prepared as side products during the synthesis of the targeted compounds as 1,1′-bis(1,3-DTF)Fc’s **8**, **10** and **11** in variable yields. Their electrochemistry was studied and compared to the previously reported derivative **9**. In CH_2_Cl_2_ on a Pt electrode and at ambient temperature, compound **12** showed two oxidation waves associated with two reduction waves with peak potentials of 698, 1158 mV at scan rates 100 mV s^−1^. In contrast the anodic peak potential (*E*^1^_pa_) in compounds **9** and **12** is higher than that of ferrocene by 171 mV for **9** and 144 mV for **12**, respectively.

## Experimental

Melting points were recorded on a Gallenkamp melting point apparatus and are uncorrected. Infrared spectra (IR) were measured on a Hitachi 260-10 spectrometer. ^1^H NMR and ^13^C NMR spectra were recorded at room temperature on a Varian Nuclear Magnetic Resonance Spectrometer (500 MHz). Chemical shifts are denoted in δ units (ppm), relative to tetramethylsilane (TMS) as internal standard, *J* values are given in Hz. MS and FAB-MS spectra were obtained using a JEOL JMS-AX505HA. CV was measured on a cyclic voltammeter (Model CS-1090/Model CS-1087). Column chromatography was performed on silica gel 60 (230–400 Mesh ASTM). Solvents were distilled before use. Acylferrocenes were prepared according to the methods that previously described in literature.

**Synthesis of 1,1′-bis(2-furoyl)ferrocene (5d).** In a dried three-necked flask, a mixture of ferrocene (3.72 g, 0.02 mol) and 2-furoyl chloride (0.02 mol) was stirred in dry CH_2_Cl_2_ (100 ml) at 0 °C for 10 min. Anhydrous aluminium chloride (2.8 g, 0.021 mol) was added at such a rate that the reaction mixture remained below 5 °C. The appearance of a blue color indicates that the reaction is occurring. This addition required ca. 20 min, and after its completion stirring was continued for 30 min with ice cooling and for a further 2 h at room temperature. The reaction mixture was cooled again in ice, 50 ml of water was added cautiously, and the resulting two phases were stirred vigorously for 30 min. After transferring the mixture to a separator funnel, the layers were separated, and the aqueous layer was extracted with two 50 ml portions of dichloromethane. The combined dichloromethane extracts were washed once with 50 ml of water, twice with 50 ml portions of 10% aqueous sodium hydroxide and dried over sodium sulfate. The dichloromethane was removed under vacuum and the residue was collected and chromatographed on silica gel using chloroform to give 0.4 g of yellow crystals of ferrocene from the early fractions followed by dark red crystals of 1,1′-bis(2-furoyl)ferrocene (**5d**) in 21% yield, mp 104–106 °C. IR (KBr) ν 3100s, 1730m, 1619s, 1606s, 1563s, 1479s, 1442s, 1375s, 1334s, 1295s, 1220s, 1155s, 1079s, 1051s, 1020s, 975s, 912s, 881s, 813s, 767s, 599s, 501s cm^−l^. ^1^H NMR (CDCl_3_) δ 7.50 (s, 2H, furan-H), 7.27 (s, 2H, furan-H), 6.54 (d, *J* = 1 Hz, *J* = 2 Hz, 2H, furan-H), 5.16 (d, *J* = 2 Hz, 4H, ferrocene-H), 4.55 (d, *J* = 2 Hz, 4H, ferrocene-H). ^13^C NMR (CDCl_3_) δ 183.60 (2 CO), 153.41 (furan, C-2, C-2′), 145.64 (furan, C-5, C-5′), 117.15, 112.13 (furan-C-3, C-4, C-3′, C-4′), 79.10 (ferrocene-C-1, C-1′), 74.23, 72.45 (ferrocene-CH). FAB-MS *m/z* (%) [M^+^ 374 (54)]. Elemental analysis for C_20_H_14_FeO_4_ (374.17), Calcd: C; 64.20, H; 3.77%. Found: C; 64.18, H; 3.72%.

**1,1′-Bis(*m*-tolylcarbonyl)ferrocene (5e)**: Similar to **5d** this was obtained as dark red crystals in 67% yield. IR (KBr) ν 3099s, 2362m, 2919m, 1639s, 1600s, 1580s, 1448s, 1398m, 1373s, 1336m, 1294s, 1224s, 1145s, 1095m, 1060s, 1031s, 890s, 836s, 781s, 752s, 669s cm^−1^. ^1^H NMR (CDCl_3_) δ 7.57 (d, *J* = 0.5 Hz, 4H, aromatic-H), 7.34 (d, *J* = 0.5 Hz, 2H, aromatic-H), 7.31 (d, *J* = 8 Hz, 2H, aromatic-H), 4.91 (t, 4H, ferrocene-H), 4.56 (s, 4H, ferrocene-H), 6.18 (s, 6H, 2 CH_3_). ^13^C NMR (CDCl_3_) δ 228.48 (CO), 138.80, 133.36, 129.30, 128.75, 126.02 (aromatic-H), 80.00 (ferrocene-C), 75.32, 73.78 (ferrocene-CH). FAB MS *m*/*z* (%) [M^+^ 422 (100)]. Elemental analysis for C_26_H_22_FeO_2_ (422.10), Calcd: C; 73.95, H; 5.25%. Found: C; 73.68, H; 5.21%.

**1,1′-Bis[(1,3-benzodithiol-2-ylidene)(2-thienyl)methyl]ferrocene (8) and 1-(2-thenoyl)-1′-[(1,3-benzodithiol-2-ylidene)(2-thienyl)methyl]ferrocene (9).** A sample of 2-(dimethoxyphosphinyl)-1,3-benzodithiole (**6**, 0.786 g, 3 mmol) was stirred in dry THF (50 ml) under a stream of nitrogen at −78 °C. A solution of *n*-BuLi (2.3 ml, 2.6 M) was added and the mixture was stirred for 15 min. The temperature of the reaction was raised to −20 °C and for further 15 min then a solution of 1,1′-bis(2-thenoyl)ferrocene (**3c**; 0.888 g, 3 mmol) in dry THF (75 ml) was added portion wise. The temperature of the reaction was raised to room temperature and the reaction mixture was kept overnight with stirring. The tetrahydrofuran was removed under vacuum and the residue was washed with water and extracted with chloroform and dried over sodium sulfate. The crude oil product was chromatographed on silica gel using chloroform/hexane mixture (1:2) to give the bis(1,3-DTF)Fc **8** as a dark red oil in the first fractions, which solidified after standing in the refrigerator to be red crystals in 31% yield, mp 171–172 °C, *R_f_* (rt, CHCl_3_/hexane 1:1) = 0.48. The polarity of the elutant was increased to be CHCl_3_/hexane (2:1) to elute a dark red solid of the corresponding Fc-DTF **9** in 12% yield.

**Data for bis(1,3-DTF)Fc 8**: IR (KBr) ν = 3060m, 1569s, 1542s, 1517s, 1448s, 1272m, 1216s, 1122s, 1031m, 813s, 732s, 698s cm^−1^. ^1^H NMR (CDCl_3_) δ 7.43 (dd, *J* = 1 Hz, *J* = 5 Hz, 2H, thiophene), 7.19 (dd, *J* = 2 Hz, *J* = 0.5 Hz, 2H, aromatic-H) 7.18–7.03 (m, 8H, 2 thiophene-H and 6 aromatic-H), 6.99–6.95 (m, 2H, thiophene-H). 4.44 (s, 4H, ferrocene-H), 4.28 (s, 4H, ferrocene-H). ^13^C NMR (CDCl_3_) δ 142.83 (thiafulvalene C=C), 136.89 (thiophene, C-2), 135.36 (aromatic-C), 127.79, 126.87, 126.04, 125.55, 125.36, 121.54, 120.62 (aromatic-CH and thiophene-CH), 113.10 (thiafulvalene C=C), 84.00 (ferrocene-C), 70.10, 68.63 (ferrocene-CH). FAB MS *m*/*z* (%) [M^+^ 678 (12)]. Elemental analysis for C_34_H_22_FeS_6_ (678.7548), Calcd: C; 60.16, H; 3.27, S; 28.34%. Found: C; 59.98, H; 3.48, S; 28.58%.

**Data for Fc-DTF 9**: The analytical data of this compound was in agreement with that previously reported in [28].

**1,1′-Bis[(1,3-benzodithiol-2-ylidene)(2-furyl)methyl]ferrocene (10).** This compound was obtained similar to the method used for **8** as an orange red oil in 62% yield, mp 98–99 °C, *R_f_* = 0.29 (CHCl_3_/hexane, 25 °C). IR (KBr) ν = 3010s, 2956m, 1646m, 1569m, 1538m, 1450s, 1286m, 1214s, 1149s, 1124m, 1014s, 923m, 754s, 667s cm^−1^. ^1^H NMR (CDCl_3_) δ 7.44 (dd, *J* = 2 Hz, *J* = 2 Hz, 2H, furan-H), 7.19–7.18 (dd, *J* = 2 Hz, *J* = 0.5 Hz, 2H, aromatic-H), 7.15–7.14 (dd, *J* = 1.5 Hz, *J* = 0.5 Hz, 2H, aromatic-H). 7.05–7.01 (m, 4H, aromatic-H), 6.52–6.51 (dd, *J* = 1 Hz, *J* = 0.5 Hz, 2H, furan-H), 6.47–6.46 (m, 2H, furan-H), 4.48–4.47 (dd, *J* = 1 Hz, *J* = 1 Hz, 4H, ferrocene-H), 4.22 (dd, *J* = 1 Hz, *J* = 1 Hz, 4H,ferrocene-H). ^13^C NMR (CDCl_3_) δ 153.30 (furan, C-2), 140.68 (furan, C-5), 136.87, 135.70 (thiafulvalene C=C), 133.20 (aromatic-C), 125.52, 125.40, 121.26, 120.83 (aromatic-CH), 113.25 (thiafulvalene C=C), 110.55, 109.12 (furan-CH), 85.98 (ferrocene-C), 69.65, 69.48 (ferrocene-CH). FAB MS *m*/*z* (%) [M^+^ 646 (12)]. Elemental analysis for C_34_H_22_FeO_2_S_4_ (646.6248), Calcd: C; 63.15, H; 3.43, S; 19.83%. Found: C; 63.12, H; 3.40, S; 19.76%.

**1,1′-Bis[(1,3-benzodithiol-2-ylidene)(*m*-tolyl)methyl]ferrocene (11) and 1-[(1,3-benzodithiol-2-ylidene)(*m*-tolyl)methyl]-1′-(*m*-toluoyl)ferrocene (12).** A sample of the 1,3-benzodithiole-2-phosphonate (**6**, 0.524 g, 2 mmol) was stirred in dry THF (60 ml) under a stream of nitrogen at −78 °C. A solution of *n*-BuLi (1.96 ml, 2.6 M) was added portion wise and the mixture was stirred for 15 min. The reaction mixture was allowed to warm to −20 °C with continuous stirring and a solution of ferrocenyl ketone **5e** (0.422 g, 2 mmol) in dry THF (50 ml) was added portion wise within 15 min. The temperature of the reaction was raised to room temperature and the reaction mixture was kept overnight with stirring. The tetrahydrofuran was removed under vacuum, the residue was washed with water and extracted with chloroform and dried over sodium sulfate. The crude oil product was chromatographed on silica gel using chloroform/hexane mixture (1:3) to give after unreacted ferrocene the corresponding 1,1′-bis(1,3-DTF)Fc **11** as dark red oils, which solidified on standing in the refrigerator in 24% yield. The polarity of the eluent was increased to be 1:2 to elute the Fc-DTF **12** as dark red semi solid material in 76% yield.

**Analytical data for 11:** Yield 24%, mp 80–81 °C. IR (KBr) ν = 3091w, 3052w, 3008w, 2915w, 1635s, 1600m, 1571m, 1548m, 1448s, 1373m, 1290s, 1222m, 1147m, 1122m, 1091w, 1051m, 744s, 667m cm^−1^. ^1^H NMR (CDCl_3_) δ 7.33 (m, 2H, aromatic-H), 7.19–7.03 (m, 4H, aromatic-H), 7.03–6.98 (m, 2H, aromatic-H), 4.35 (s, 4H, ferrocene-H), 4.24 (s, 4H, ferrocene-H), 2.37 (s, 6H, 2CH_3_). ^13^C NMR (CDCl_3_) δ 142.59, (aromatic-C), 137.24, 135.72 (thiafulvalene C=CS_2_), 128.97, 128.82, 128.42, 126.40, 125.89, 125.40, 125.17, 121.41, 120.62 (aromatic-C and CH), 86.81 (ferrocene-C), 69.79, 68.58 (ferrocene-CH), 21.53, 21.99 (2 CH_3_). FAB MS *m*/*z* (%) [M^+^, 694 (18)], EI MS *m*/*z* (%) [M^+^ 694 (100)]. Anal. Calcd. for C_40_H_30_FeS_4_ (694.06), Calcd: C; 69.15, H; 4.35, S; 18.46%. Found: C; 69.23, H; 4.21; S; 18.21%.

**Analytical data for 12:** Yield 76%, mp 68–69 °C. IR (KBr) ν = 3056m, 2958s, 2925s, 2869m, 1693s, 1600m, 1571s, 1548m, 1448s, 1216m, 1122s, 1089m, 1031m, 740s, 667s cm^−1^. ^1^H NMR (CDCl_3_) δ = 7.66 (q, *J* = 0.5 Hz, 2H, aromatic-H), 7.30 (m, 3H, aromatic-H), 7.23 (m, 1H, aromatic-H), 7.18 (m, 1H, aromatic-H), 7.07–7.01 (m, 5H, aromatic-H), 4.93 (t, *J* = 2 Hz, 2H, ferrocene-H), 4.59 (t, *J* = 2 Hz, 2H, ferrocene-H), 4.37 (t, *J* = 2 Hz, 2H, ferrocene-H), 4.24 (t, *J* = 2 Hz, 2H, ferrocene-H),), 2.38 (s, 6H, 2CH_3_). ^13^C NMR (CDCl_3_) δ 198.95 (CO), 142.17 (aromatic-C), 139.79, 138.80 (thiafulvalene C=CS_2_), 137.98, 136.87, 135.71, 132.25, 129.82, 129.36 (aromatic-C), 128.97, 128.78, 128.73, 128.61, 127.96, 126.25, 125.64, 125.46, 125.34, 122.39, 121.48, 120.77 (aromatic-CH), 88.18 (ferrocene-C), 78.32 (ferrocene-C), 74.18, 72.20, 70.88, 69.03 (ferrocene-CH), 21.54, 21.44 (2 CH_3_). FAB MS *m*/*z* (%) [M^+^ 558 (100)]. Anal. Calcd. for C_33_H_26_FeOS_2_ (558.08), Calcd: C; 70.96, H; 4.69, S; 11.48%. Found: C; 70.87, H; 4.71; S; 11.50%.
